# A benchmark dataset for printed Meitei/Meetei script character recognition

**DOI:** 10.1016/j.dib.2022.108585

**Published:** 2022-09-15

**Authors:** Yanglem Loijing Khomba Khuman, Salam Dickeeta Devi, Ch. Ponykumar Singh, H. Mamata Devi, N. Ajith Singh

**Affiliations:** a Department of Computer Science, Manipur University, India; b Department of Computer Science, South East Manipur College, India

**Keywords:** Meitei/Meetei script, Optical character recognition, Natural language processing, Printed document images

## Abstract

The Manipuri language is the official language of the Indian state of Manipur. The language belongs to the Tibeto-Burman family of languages. A benchmark Meitei/Meetei script printed document images dataset is presented in this article. The dataset contains raw 824 pages of printed documents and binarized images, text files, and XML files for each raw image. It also includes 51,460 isolated character samples, composed of 27 consonants, 7 half-consonants, 8 vowels, and 10 numerical. This dataset could be used for optical character recognition (OCR) research and in the different research areas of natural language processing (NLP).

## Specifications Table

**Table:** 

Subject	Computer science

Specific subject area	Optical Character Recognition, Image Processing, Machine Learning, Natural Language Processing.
Type of data	Scanned document images Text files Annotated files for images
How the data were acquired	Documents of different books, magazines, and newspapers are scanned using Canon Flatbed scanner “CanoScan 4400F”. We used the “Aletheia (Document Analysis System)” tool to annotate scanned documents, developed by Pattern Recognition and Image Analysis Research Lab, University of Salford, Manchester.
Data format	.tif .txt .xml
Data source location	• Institution: Department of Computer Science, Manipur University • City/Town/Region: Canchipur, Manipur • Country: India
Data accessibility	Data presented in this article is freely available at: Repository name: Mendeley Data Data identification number: DOI: 10.17632/rw4b2zdk95.2 Direct URL to data: https://data.mendeley.com/datasets/rw4b2zdk95/2

## Value of the Data

This data is beneficial for training the machine learning model for character recognition.

**Fig. 1:**
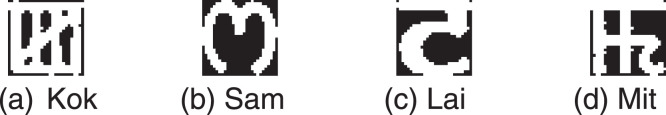
Some samples for isolated characters 28 × 28 px.

• It is useful in character recognition and can also be used in various research areas of Natural Language Processing.
• This is only annotated dataset for printed Meitei/Meetei script document images.
• It also includes isolated characters composed of consonants, half-consonants, vowels, and numerals, all in one place, as shown in Table 1, Table 2, Table 3, Table 4 and Fig. 1.

**Table 1:** Meitei/Meetei Script consonant.

**Fig. 4:**
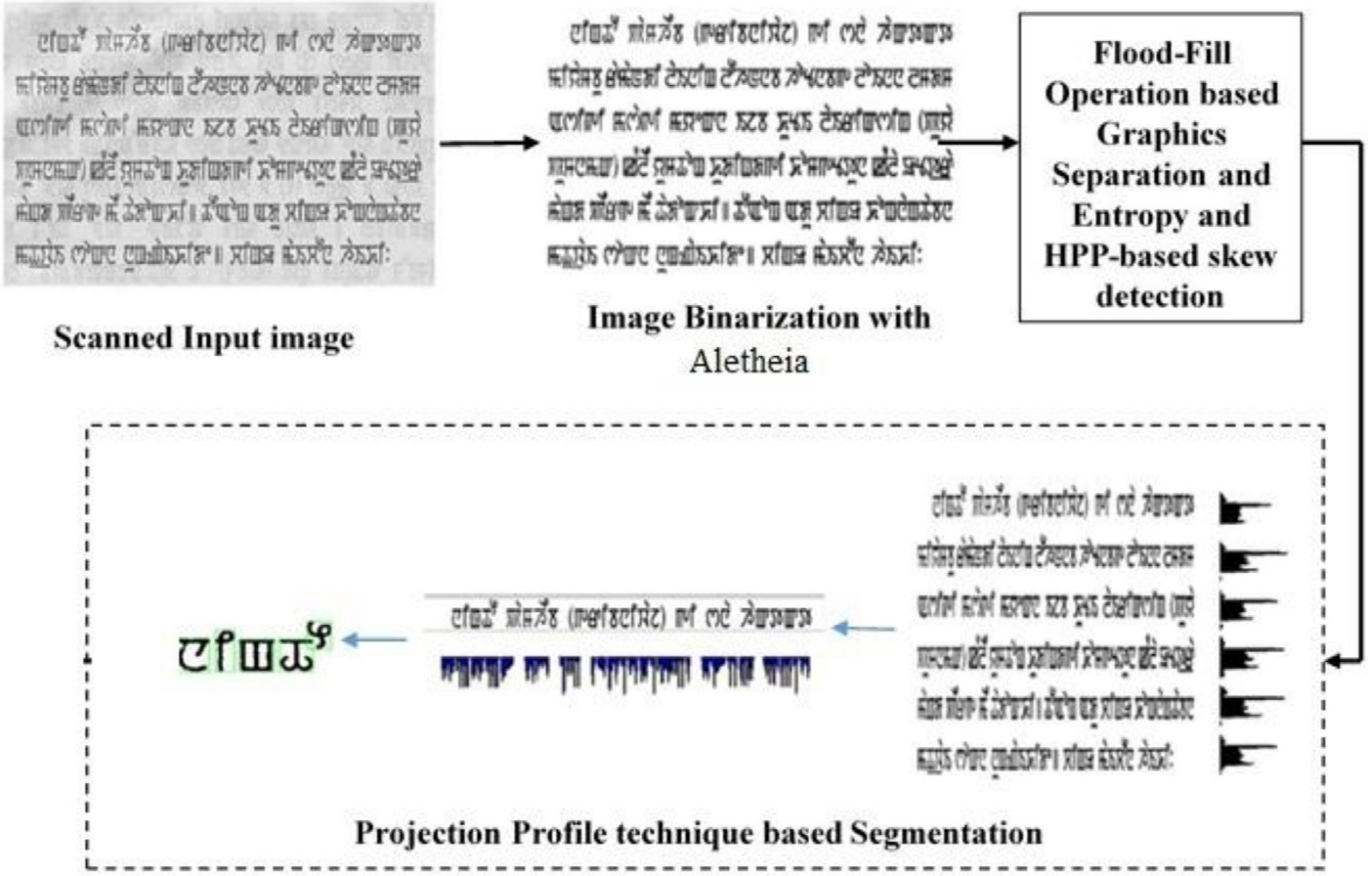
Various processes of image pre-processing.

**Table 2:** Meitei/Meetei script half-consonant.

**Table 3:** Meitei/Meetei Script vowel.

**Table 4:** Meitei/Meetei script numerals.
• Isolated characters are stored in a separate folder for each character and the folders' names are in Unicode decimal values, so it is more convenient while training the machine, as shown in Table 5.

**Table 5:** Meitei/Meetei script Unicode values.

**Table 6:** Brief description of the books.

Book Code Used in the Dataset	Original Title	Translated Title in English	Class of Book	Brief Description of the Book	Number of scanned pages
BN_BOOK_1	Wahoulol khutpai	Language Guide	Grammar	This book is about the Meitei/Meetei language grammar. It covers the essential rules of the language.	87
BN_BOOK_2	Elisha amagi Mahao	The Taste of an Hilsa	Story	It is a story about a low-income family's undesirable physical and mental environment. It depicts the unwilling human compromise with the present social system and, at the same time, find out the latent reasons behind the corruption and bribery.	87
BN_BOOK_3	Hannuba Hannubi pal thaba	Old Man and Old Woman planting Taro	Folk Tale	This folk tale is about an old couple being cheated by some monkeys while planting taro plants. After being cheated, the couple got angry and took revenge on the monkeys.	58
BN_BOOK_4	Tharon	Sequence of Months	General Knowledge	This book explains the lunar calendar used by the Meitei people of Manipur for their religious and agricultural activities.	55
BN_BOOK_7	Sennahoudraba mikupta.	Unattended Moment	Short Story	This book contains 17 short stories written in pure Manipuri Language.	91
BN_BOOK_8	Liksangnu Saphabi	Liksangnu Saphabi	Historical fiction	This book is about beautiful and intelligent Liksangnu saphabi, the only daughter of a village chief named Thangal Khulakpa. It's a story that took place during the Phakhangba Era.	59
BN_BOOK_10	Miyamgi Ehou-2001 June 18-Thawai 18	People Uprising-2001 June 18 - 18 Souls	Biography	This is a biography of 18 persons who laid down their lives to protect the integrity of the Manipur State on June 18, 2001.	6
BN_BOOK_11	Paabiyu Paaraga Khanggani	Read then you will know	Linguistic	It is a collection of Phrases, Quotes, Proverbs, Acronyms, etc. of the Manipuri language	57
BN_BOOK_15	Nahagi wahang-Ahal gi paokhum amasung Khambagi wahang – Nongbal gi paokhum	Youth's question – Elder's Answer and Khamba's question – Nongbal's Answer	Knowledge	Collection of questions on various activities in our lives and their answers.	86
BN_BOOK_16	Erat Thouram Thounirol Larik	Ceremonial Rituals Book	Rituals & Practices	It is a collection of rituals followed in traditional Manipuri ceremonies like marriage, weaning, funeral, etc.	134
BN_BOOK_20	Saranggi Nongsa	Lion in a Cage	Poem	Collection of Various Poems	3
NEWS_PAPER	News Paper	NA	NA	News columns are collected from daily locals (The Sangai Express, Poknapham, Hueiyen Lanpao) published in Meitei/Meetei Script.	101

**Fig. 2:**
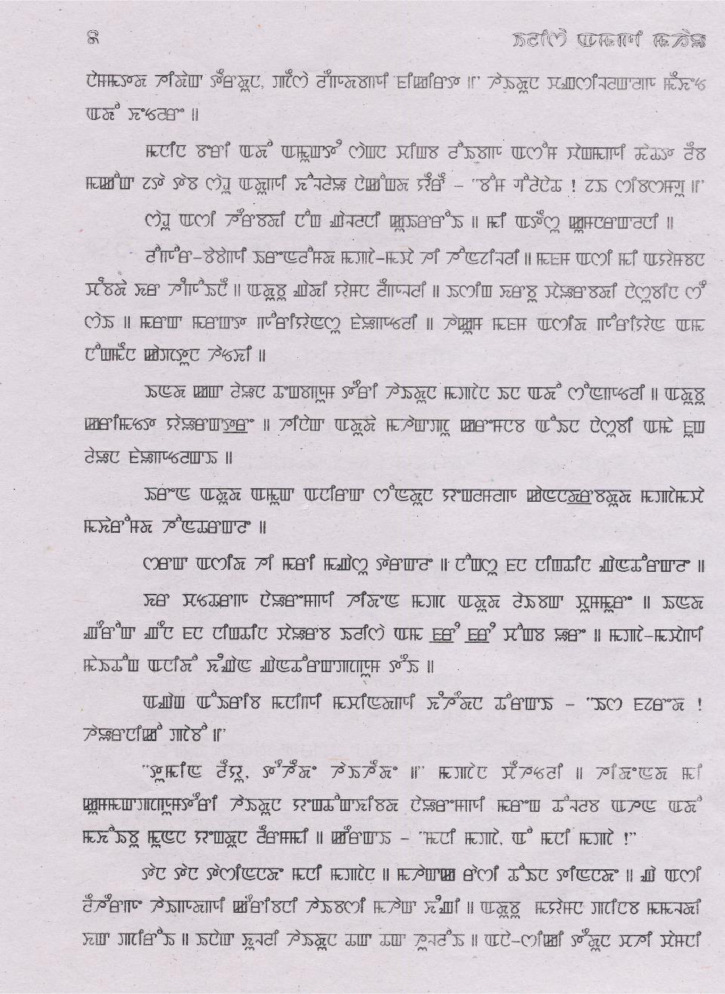
Raw scanned image (R_BOOK_2_PAGE_8).

**Fig. 3:**
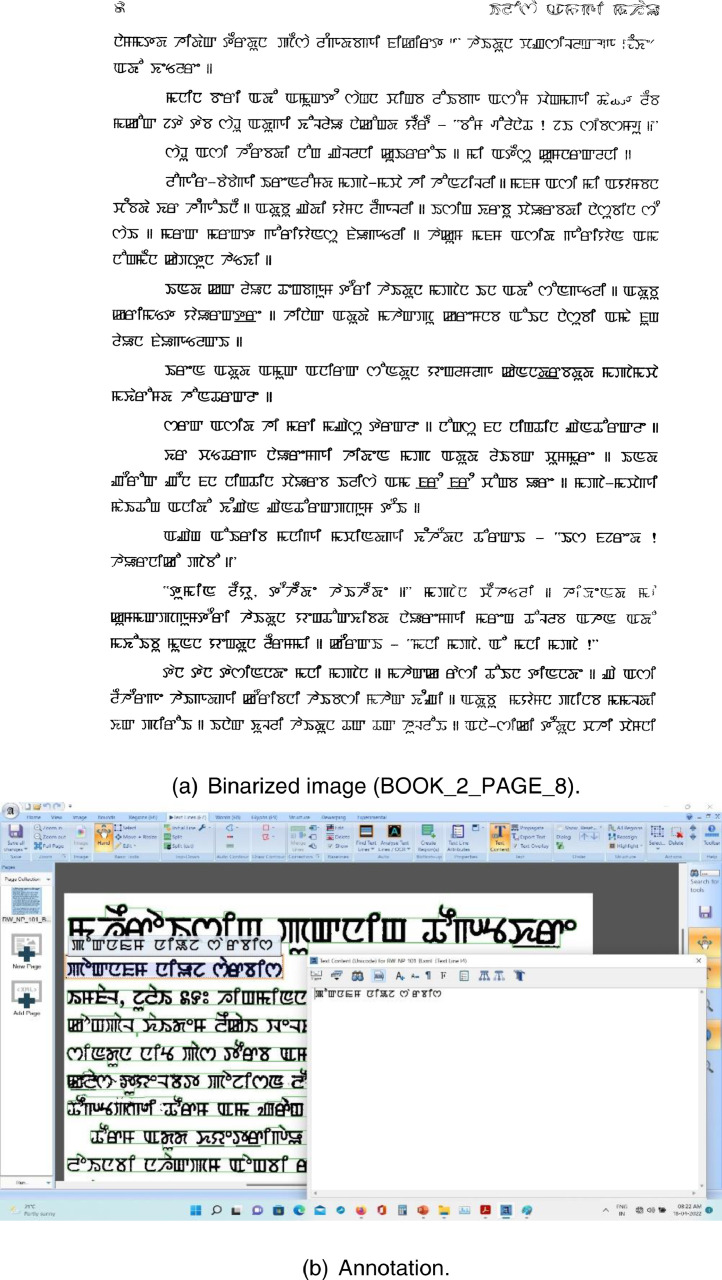

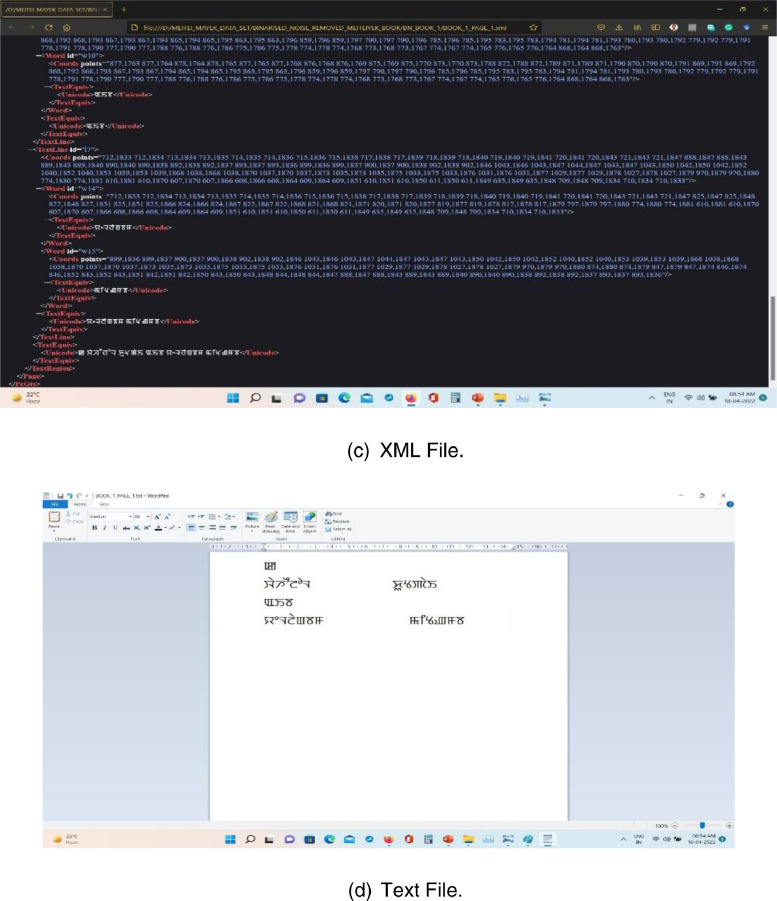
Binarization and annotation using Aletheia.

## 1 Data Description

### 1.1 A Brief History of Manipuri (Meitei/Meetei) Script

Manipuri is one of the official languages of the Indian state of Manipur in north-east India and has about 1.6 million speakers. It is a member of the Tibeto-Burman branch of the Sino-Tibetan language family. The Manipuri (also called Meiteilon) is spoken in the Indian states of Manipur, Assam, Nagaland, Tripura, and some parts of Bangladesh and Myanmar. The language was written using the Meitei/Meetei script until the early 18th century CE. Bangla script was used to write the language during the last three centuries.

Following the revival, the Meitei/Meetei script is slowly reintroduced as the primary script for writing Manipuri. The adoption of a modernized writing system in educational institutions was authorized by state legislation in Manipur in 1980. In 2009, it was encoded using Unicode . This script uses bodily parts to name the letters is one of its distinctive elements. For instance, the first letter "Kok" stands for "head," the second letter "sam" for "hair," the third letter "lai" for "forehead," etc. The holy book " Wakoklol Heelel Theelel Salai Amailol Pukok Puya" corroborates this, which explains how each script came to get its appellation.

## 2 Experimental Design, Materials and Methods

We have scanned 824 pages of document images using “CanoScan 4400F” scanner from different books, magazines, and newspapers. For image binarization and annotation, we used the “Aletheia (Document Analysis System)” tool, developed by Pattern Recognition and Image Analysis Research Lab, University of Salford, Manchester, as shown in Fig. 2, Fig. 3.

The extraction steps for an isolated character are as follows:

• Scanned document images are binarized using the “Aletheia (Document Analysis System)” tool.
• Skew is detected and corrected using [1].
• Graphics images are removed using [2].
• We use horizontal projection, vertical projection, and connected component analysis for lines, words, and character segmentation [3]; the extracted isolated characters are of 28 × 28 px size.

## CRediT authorship contribution statement

**Yanglem Loijing Khomba Khuman:** Conceptualization, Methodology, Software. **Salam Dickeeta Devi:** Data curation. **Ch. Ponykumar Singh:** Writing – original draft. **H. Mamata Devi:** Supervision, Writing – review & editing. **N. Ajith Singh:** Software, Validation.

## Declaration of Competing Interest

The authors declare that they have no known competing financial interests or personal relationships that could have appeared to influence the work reported in this paper.

## Data Availability

A Benchmark dataset for printed Meitei/Meetei script character recognition (Original data) (Mendeley Data).
